# Combining Multi-Agent Systems and Wireless Sensor Networks for Monitoring Crop Irrigation

**DOI:** 10.3390/s17081775

**Published:** 2017-08-02

**Authors:** Gabriel Villarrubia, Juan F. De Paz, Daniel H. De La Iglesia, Javier Bajo

**Affiliations:** 1 Faculty of Science, University of Salamanca, Plaza de la Merced s/n, 37002 Salamanca, Spain; fcofds@usal.es (J.F.D.P.); danihiglesias@usal.es (D.H.D.L.I.); 2 Department of Artificial Intelligence, Universidad Politécnica de Madrid, Campus Montegancedo s/n, Boadilla del Monte, 28660 Madrid, Spain; jbajo@fi.upm.es

**Keywords:** ambient intelligence, wireless sensor, fuzzy logic, smart irrigation, virtual organizations of agents

## Abstract

Monitoring mechanisms that ensure efficient crop growth are essential on many farms, especially in certain areas of the planet where water is scarce. Most farmers must assume the high cost of the required equipment in order to be able to streamline natural resources on their farms. Considering that many farmers cannot afford to install this equipment, it is necessary to look for more effective solutions that would be cheaper to implement. The objective of this study is to build virtual organizations of agents that can communicate between each other while monitoring crops. A low cost sensor architecture allows farmers to monitor and optimize the growth of their crops by streamlining the amount of resources the crops need at every moment. Since the hardware has limited processing and communication capabilities, our approach uses the PANGEA architecture to overcome this limitation. Specifically, we will design a system that is capable of collecting heterogeneous information from its environment, using sensors for temperature, solar radiation, humidity, pH, moisture and wind. A major outcome of our approach is that our solution is able to merge heterogeneous data from sensors and produce a response adapted to the context. In order to validate the proposed system, we present a case study in which farmers are provided with a tool that allows us to monitor the condition of crops on a TV screen using a low cost device.

## 1. Introduction

Drought, climate change and pollution subject our water resources to big changes, and as the situation gets worse with time, more people experience its negative effects; currently, four out of every 10 people in the world are affected by a lack of water. Our population continues to grow and with it our needs for more water grow, for both industrial and domestic purposes. Our work focuses on optimizing water management in the agricultural sector, being the largest economic sector in the world. It is estimated that the agricultural industry wastes 60% of the 2.500 billion litres of water used each year [1,2,3]. In comparison to the current crop irrigation systems, we seek a more economic and effective solution that incorporates intelligence and context-awareness. This is possible due to the remarkable progress that has been made in the field of electronics in the last decade, whereby the size of end devices has decreased and their production costs as well. As a result, a variety of low cost sensors and communication devices is now available, allowing us to propose new solutions that can solve many every day challenges in an economic way. The possibility of using sensor networks in the agricultural sector would allow us to acquire data and look for intelligent solutions, helping us to create a system that ensures proper crop growth and optimizes water usage. However, the current monitoring systems do not incorporate a minimum degree of intelligence and do not have the ability to adapt to the environment. Moreover, the implementation of industrial equipment for the control of crop irrigation is hindered by its high cost and complexity; the lack of such equipment on farms results in unnecessary water wastage.

A multi-agent system (MAS) [4,5] is the most suitable option for this solution. This is because a MAS includes an Intelligent Distributed Artificial System, which incorporates social aspects and human reasoning to solve problems. Specifically, this work proposes an architecture based on virtual organizations through the use of a multi-agent open source platform called PANGEA [6], which incorporates services that allow sensor networks to be interconnected. This design makes it possible for agent societies to include organizational aspects of human societies, improving self-regulation and self-organization. The use of autonomous agents embedded in devices with limited computing capabilities makes the PANGEA platform a perfect candidate for the planned system. Each agent may represent an autonomous entity that consumes and provides different services. Collaboration between agents from the same organization offers more distributed and complex functionalities. One of the challenges that must be faced in this work is the fusion of information that comes from heterogeneous sources; the aim is to design an agent type that fuses heterogeneous data and obtains the crops’ growth patterns. The proposed system should guarantee growth and scalability of the platform.

The assignment of roles to virtual organizations of agents will ensure system flexibility and will provide us with the ability to add new functionalities, creating a completely transparent layer for the applications that are developed for the user. The communication between the different components of the system must be efficient and low power consuming, since it must be capable of operating without direct sunlight. The proposed system must be able to monitor a crop and automatically supply the amount of water needed, this will be done through a predictive model with input variables regarding the climate, humidity of the subsoil, the force of wind, sunlight, temperature and the time of the day. Fields generally have very vast areas of land and sensors often have to be installed at large distances from one another, this is why a wireless connection between them is necessary. To this end, a network of autonomous sensors has been designed, with manageable battery consumption, which coordinates and generates different events produced in the system. Wireless Sensor Networks (WSNs) [7] are used to analyse environmental behaviour and the existing interaction between different sensors, they make decisions automatically on a daily basis [8].

The key novelty of the system presented in this paper is the use of fuzzy-logic and its application in a multi-agent environment. Those agents interact autonomously giving the system greater flexibility and intelligence. We also describe how the open-hardware platform called “Open Garden” is used; it has been designed specifically for the collection of essential agricultural data.

In addition, new interfaces have been implemented. Among these interfaces, the described system is a pioneer in using television as a data representation interface. This is very important because it is one of the devices most commonly used by final users. We should also point out that it is a low cost system, this enables small and medium-sized farmers to access this technology without having to invest a large sum of money.

This article is organized as follows: Section 2 reviews the state of the art and related projects, Section 3 describes the proposed architecture, Section 4 describes the case study conducted to evaluate the system, and finally Section 5 presents the results obtained and our conclusions.

## 2. Background

Currently, a variety of automatic crop irrigation systems are available in the literature, [9,10] and this section provides a detailed analysis of them. Existing solution are limited by various factors: their design does not adapt to the particular requirements of different crop species; the quantity of water supplied by each manufacturer is different, making it difficult to determine the exact amount of water to be supplied to a crop. Moreover, irrigation systems are extremely expensive and are provided by their manufacturers with closed architectures that restrict customization or inter-compatibility with other devices. For example, it is not possible to interconnect sensors from different manufacturers or to integrate data in an application that could be controlled from a smart TV.

The current issue of wasting natural resources has called the European Union to action. The EU is now encouraging the development of solutions that ensure ecological efficiency. Some of the best known projects that have been funded by FP7 [9] include the following:

WATERBEE DA (REF: 283638 Funded FP7-SME): this system allows farmers to save water by watering only at the time and place required [11]. Financed by European Community funds, the project team developed a prototype of a sustainable irrigation system. The tests showed savings of 21%, registering peaks of up to 44%. The impact of irrigation was also reduced to 23%. This system features wireless communication and environmental sensors, providing intelligent, flexible, easy to use, affordable and accurate programming. Moreover, this system can be adapted to the specific requirements of each user, the humidity of the soil and environmental conditions, and to different agricultural management systems [9,12,13]. Related works such as [14] include a mobile application that has been used to manage irrigation with pivot in the state of Colorado (USA), while another work in Florida uses evapotranspiration-based irrigation controllers [15] to define schedules. Some works such as [16,17] operate with a Smart Irrigation Decision Support System; these systems include machine learning techniques such as artificial neural networks, fuzzy decision systems to analyze the water in the soil, or to establish previous irrigation patterns. However, these types of supervised systems require previous expert knowledge to train the algorithms.

OPTIFERT (REF: 2836772 Funded FP7-SME): is based on an innovative automatic irrigation system for medium and large scale agricultural holdings [18,19]. This system combines fertilization and irrigation, and reflects the increasingly widespread trend among farmers to use computers, making it easier to keep track of the consumption of water and fertilizer. The system is composed of a soil sensor, a data processor, a control and distribution unit that monitors fundamental parameters of soil, and plant requirements in real time. The control software is able to access databases containing information about crop growth and relate it to crop species and soil (type, structure and fertility) data, as well as economic data on costs and prices. It is also possible to get weather forecasts and insert them into the system. In addition, the user can add data, such as reports on crops and planting times. It can obtain data that determine the right amount of water and fertilizer for each stage of crop growth.

ENORASIS (REF: 282949 Funded FP7-SME): by using this system, farmers can install a network of wireless sensors on their farms to gather information on factors affecting the crops’ need for water, including soil moisture, atmospheric temperature, insolation, wind speed and precipitation [20,21]. The system also has a set of valves to measure any increase in the amount of water. This solution saves water, prevents soil erosion and generates both environmental and economic benefits. This system also uses a weather forecasting model that combines satellite images from the fields and the information from sensors to create a specific meteorological prediction. The model offers such a fine resolution that predictions can focus on areas of up to two square kilometres. Moreover, crop data can be used to prepare a watering plan, allowing the farmers to decide if they need to add more water to the ground [21].

IRRIMAN LIFE (funded program Life+): granted in 2015, the project is based on an automated system. Using an algorithm [19,22,23], irrigation needs are determined according to the water contained in the soil, the plant, and the atmosphere, all of which are measured on a continual basis using different sensors in the endometrial system. The project ensures the efficient use of water resources, the improvement of the quantitative management of water, and the preservation of the high quality level of water, and avoiding the misuse and deterioration of water resources. This is a very interesting project which has recently started [24].

This section has provided clear initiatives [13,25] to implement solutions that combine different sensors for the purpose of using natural resources in a more efficient way e.g., rationing electric power employed by the irrigation equipment or rationing limited resources, such as water. While these systems are composed of different sensors, they use closed platforms and lack the capacity to interact with external agents. Moreover, they lack the intelligence that equips them with learning and adaptation capabilities. Consequently, we need an open and heterogeneous platform that allows us to merge information from all the sensors for subsequent analysis and study.

Having begun as recently as 2014–2015, these projects are still in the development phase. Their use on conventional farms requires a significant investment, making them appropriate only for large areas. Extrapolating these systems for use on smaller areas, such as a small vegetable garden or greenhouse, or using them simply to monitor a crop during a short period of time, would make the cost of acquiring the necessary equipment far too expensive for most farmers.

Nevertheless, a comparative study of commercial solutions has been carried out for small scale farms. The solutions that incorporate sensors do not include systems based on fuzzy logic which allow to establish the watering quantities in a precise way. Aifro WaterEco [26] considers climatology in order to lower or increase irrigation but it is focused on the definition of threshold values and does not include fuzzy logic or sensors, such as soil and land humidity. Blossom [27], encompasses crop irrigation and generation of calendars, depending on the climate these calendars can be edited manually, it has common functionalities but allows for remote management, it also does not include fuzzy logic in its behavior. BlueSpray [28] includes seasonal information to adjust irrigation as in the previous example, it does not include fuzzy logic based behavior. GreenIQ [29] and IrrigationCaddy [30] are conventional programs that can be managed remotely from mobile applications and include the feature of creating irrigation calendars. Lono [31] incorporates threshold values and seasonal information and reduces crop watering according to the thresholds, as in the previous cases it does not include fuzzy logic and does not have weather sensors.

On the other hand, the Orbit B-Hyve [32] system incorporates a control through smartphones that is able to change some parameters in order to edit the system schedule. The parameters that device takes into account when configuring the irrigation timer are: the slope of the site, the soil type, if it is in the sun or shade, history of rainfall in the area and the current weather. The Rachio Smart Sprinkler Controller [33] system also has a Wi-Fi connection and is able to send the data from the sensor to the user’s smartphone. This device requires an initial configuration which is established by indicating the type of crop and the type of soil. In this way, the system can estimate the irrigation time required by the crop. The fuzzy system is not applied, nor are the flexible rules. Rainmachine [34] is another commercial system which incorporates an automatic irrigation program. It is capable of calculating the percentage of evaporation and transpiration of the soil, according to the weather conditions obtained from the data of the meteorological service. This system, like the others, does not include fuzzy knowledge. The Spruce irrigation [35] system combines the information obtained from all the temperature and humidity sensors and rainfall forecasts. Lastly, we list the Raincommander [36] system for its ease of use and its integration with mobile devices for remote irrigation control. However, this system lacks an intelligent configuration, it has no fuzzy logic rules, and only considers the schedule and the irrigation time that has been configured manually by the user.

After a careful review of the related literature, this work focuses on a novel design of an open architecture composed of virtual agent organizations. The proposed system is economic and can be customized to fit the needs of each farmer making it possible to monitor and automate the irrigation of any crop species. From an analytical point of view, it will be necessary to store the information of each sensor in a remote database, this will allow farmers to examine the effectiveness of the system. Finally, we can deliver these functionalities to the user as services; users will be able to control irrigation from a TV screen, using a remote. In conclusion, the major novelties of this work are: (a) the ability to estimate irrigation time through the use of multi-agent virtual organization technology that executes a fuzzy algorithm, (b) the deployment of agent models in devices with limited capabilities using the PANGEA architecture, (c) the monitoring and control of the irrigation system with a TV remote (thanks to the use of wireless sensors networks).

## 3. Proposed Architecture

**Figure 1:**
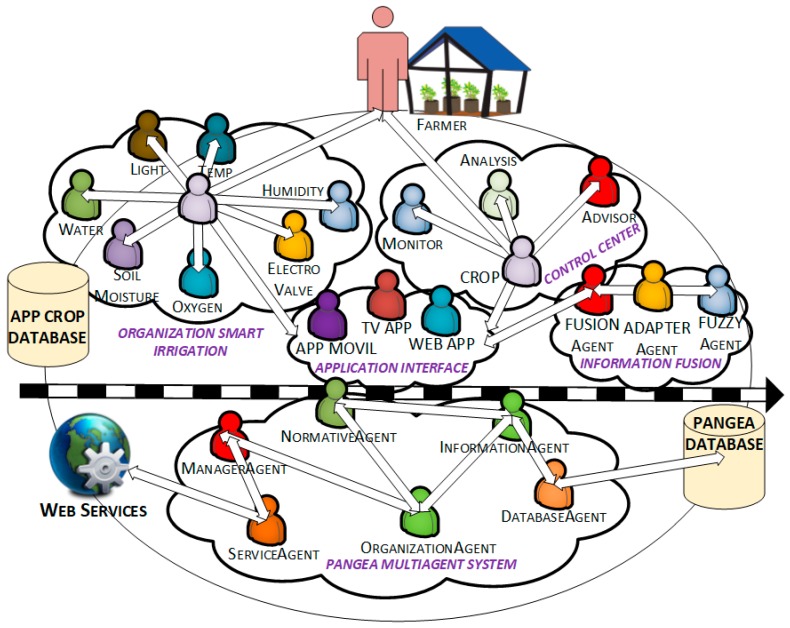
Agent organization flow chart.

In the field of computer science and artificial intelligence the use of multi-agent systems deals with the interconnectivity of intelligent agents that collaborate together to solve a complex problem. The use of a combination of agents in wireless sensor networks allows for the design of new platforms with advanced computing capabilities. The design of a multi-agent system based on virtual organizations allows one to monitor and control an irrigation system. The different algorithms that make up the case study should be embedded in embedded devices like sensors or small microcontrollers. To achieve this, we have chosen a multi-task architecture that makes it possible for virtual organizations to have a dialogue between them, this architecture makes up the case study since distributed processing techniques can also be used with it. The proposed architecture must be dynamic, have the ability to merge information from heterogeneous data sources, and contain advanced analysis and prediction capabilities. The dynamism that a multi-agent architecture offers allows us to add new sensors, adapting them to the requirements of the environment. One of the main innovations of this architecture is a design based on organizational theory, which can both imitate and collaborate with human organizations related to crop irrigation. This Section will present the design of an architecture that (1) allows for the creation of an open and self-organizing system, and (2) can handle different types of sensor networks, thus facilitating the dynamic addition of new protocols based on the emergence of new technologies such as Zigbee, RFID, Wi-Fi, and Bluetooth. We will explain the design of the architecture in detail, as well as the agents that make up each virtual organization, as shown in Figure 1.

The architecture is composed of two distinct parts: the bottom is formed by the minimum agents that make up the multi-agent PANGEA system; the top consists of different virtual organizations on which this case study is based, and whose operation is explained below:

*Organization Information Fusion*: This refers to an organization whose objective is to merge the information provided by the sensor networks (lower layers), which is then integrated with the virtual agent organizations (upper layers). In this organization, agents emulate the human behaviour of adding environmental information, thus making it possible to obtain far more advanced knowledge than what is generally provided by individual data. Also, the information formats controlled by each sensor are transformed to a common and manageable standard for all architecture. The internal message protocol chosen for the communication between agents of the platform is a messaging protocol of plain text that is based on the standard RFC1459 [37].

*Organization Smart Irrigation*: This refers to an organization that is in charge of extracting and collecting information from different sensors. Its main function is to transform the physical layer data so that they can be used by other agent organizations. Each agent communicates in a unidirectional way with a central officer who organizes and manages the communication. In this organization, there are two different roles: one held by officers, who obtain the values of the sensors; and another secondary role, in the coordination of tasks and communication with other organizations of the architecture. The different agents that form part of this organization are shown in Table 1. These agents are deployed in the nodes to extract information from the environment, the obtained data are sent to the central node which sends them to the main server.

*Organization Control Center*: This organization is responsible for monitoring information obtained by agents, and belongs to the *Smart Irrigation Organization*. The most important task is the intelligent analysis of information and prediction based on the data collected from different sensors. The Crops agent is in charge of coordinating monitoring tasks, analysis and alerts, additionally this agent is responsible for managing the defined rules for each type of crop. In the case of an anomaly, an alerting situation, or a value outside of the usual range, this organization will be responsible for initializing the process of resolving the anomaly, which is then notified to the system administrator.

*Organization Application Interface*: This organization is in charge of adapting data from the other virtual organizations, and then representing this data in the application layer. As the organization is an interface, the applications inside the client can easily interact with the platform. For example, in the case of an external device that has to request a particular functionality from the system, or any application such as “Web Application or Smart TV”, the data have to be adapted from the raw data to a standard format. This organization will develop an adaptation function, also known as the connector, for later use in any application. The presented case study has several connectors or gateways whose main function is to transform data from the architecture so that the data can then be represented on a smartphone, a Smart TV or a web application.

*Pangea MultiAgent System*: The decision to use PANGEA was based on its ability to create virtual organizations, which are characterized by their dynamic nature. This is the most singular feature, since other alternatives, such as THOMAS or JADE are not dynamic. PANGEA is a cost-free, multi-agent framework developed by the BISITE research group and anyone can use it. The PANGEA architecture can function with devices with limited computing capabilities, this feature is a big advantage because it enables us to deploy agents embedded in hardware. The fact that sensors are powered by sunlight makes this feature even more essential for the system. Moreover, limited computing capabilities are necessary for the algorithms responsible for data processing, as well as for efficient communication between the sensors in the system. The agents specialized in the management of virtual organizations are defined in [38], these agents are responsible for managing the agents inside the whole virtual organization. Below, we focus on the basic functions of the agents that manage the virtual organization executed within PANGEA.

- *DatabaseAgent*: This agent plays a storage role in the organization to provide persistence to the information in the organization. It is the only agent with database access privileges. Its objective is to perform backup tasks, as well as to ensure the correct consistency and storage of information. This agent communicates with the rest of the agents in the organization.
- *Information Agent*: This agent manages the services inside the virtual organization. It is also known as the “yellow pages” agent, as it allows other agents to publish the services provided, so that others can access them. When a new device or application uses the architecture for the first time, the corresponding agent should consult the specific services offered in the virtual organization.
- *Normative Agent*: One of the most important aspects in a virtual organization are the norms that govern the organization. This agent is responsible for the security when establishing communication between devices. When an application uses a specific functionality, this agent is in charge of checking whether it is authorized to do so, using a rules engine based on DROOLS [39,40].
- *Service Agent*: This agent distributes functionalities as web services. It is also a gateway to communicate external web services outside the system with the agents in the organization. To encourage greater abstraction, functionalities, and different capabilities offered by the architecture, some services are exposed; this mode favors greater integration independent of programming languages.
- *Manager Agent*: This agent is responsible for performing periodic system management, verifying if there is any overloaded functionality, and ensuring free of errors communication between people and organizations.
- *Organization Manager*: This agent plays a very important role in the architecture given that it is responsible for verifying the operation of all the virtual organizations, dealing with security, and balancing and providing encryption of the frames between the most important agents.

In the APP Crop Database different information is included, such as the irrigation rules liked to the type of crop. In these rule we include information on the geographic location, this data base is synchronized with a central server to ease the addition of new crops.

## 4. Monitoring Platform and Irrigation

This Section presents a case of study of a small crop environment combining a low cost hardware and multi-agent systems, which allows the fusion of information captured by different sensors. The chosen hardware platform is called the OpenGarden. Due to the wide variety of crops and the source that we can monitor, the architecture can be implemented in three different scenarios: indoors (houses and greenhouses), outdoors (gardens and fields), and with hydroponic agriculture (plants in water-based facilities).

**Figure 2:**
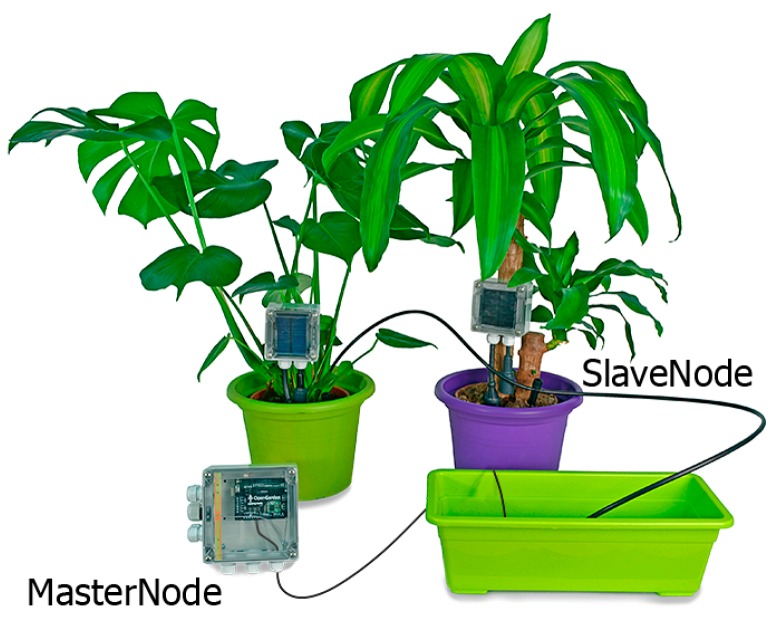
Architecture sensor network.

The system must provide the ability to control the state of the plant through the detection of several parameters: moisture in the ground, humidity, brightness sensors for pH, conductivity, temperature, oxygen, and water level. The topology between different sensors is represented in Figure 2. There is a slave node for each type of crop or plant to be monitored, and a single central node that connects the cultivated area. There are two types of nodes: slave nodes, which send the information from the interconnected sensors; and a central or primary node, which acts as a gateway sending data to an agent that resides on a web server, using existing wireless technologies (Wi-Fi, GPRS, 3G).

**Figure 3:**
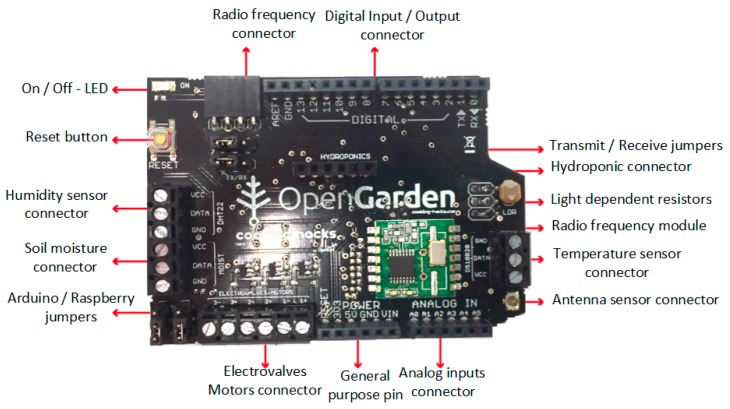
OpenGarden Gateway Node functionality board [42].

Below is a detailed explanation of the hardware used in this solution. The Gateway is powered by an Arduino-one controller [41]. The slave nodes send information to the central node via network, with a star topology for the transmission of information, using an Amplitude-Shift Keying (ASK) modulation. The selected band frequency is 433 MHz, due to the autonomy of the devices and the need for efficient communication, where the quantity identity of data shared between the different nodes is not very high. The gateway node is composed of an OpenGarden Shield [42]. The number of central nodes varies depending on the size of the farm, independent subzones can be established with different configurations. The distance between the central zones depends on the visibility of the environment. Using a 433 MHz range we attained interconnectivity between the nodes at a distance of 250 linear meters with total visibility. The functionality offered by each of the controller pins is shown in Figure 3.

The shield of the master node allows us to technologically connect different types of sensors and to gather information from any sensor that is available on the market. In addition, it ensures interconnectivity with external hardware as Arduino or Raspberry by using a serial port. Of note, the shield incorporates a battery for autonomous operation which uses sunlight to power itself during the day. The controller is based on a DS1307 chip to time programming. It has an I2C interface that allows the interconnection of virtually any sensor currently on the market, and an accurate clock that will adjust to time changes. It can detect if there is a fault in the electrical circuit, and consumes less than 500 nA. The central node is capable of expanding its functionalities, providing us with the possibility of adding any type of sensor or functionality that we might need. This expansion port consists of 12 pins (analog and digital) which allow to, for example, adapt the system for activating monitoring systems, monitoring the condition of motors and pumps or if we want to use the system in greenhouses; to control the ventilation system, airflow and motorized doors.

**Figure 4:**
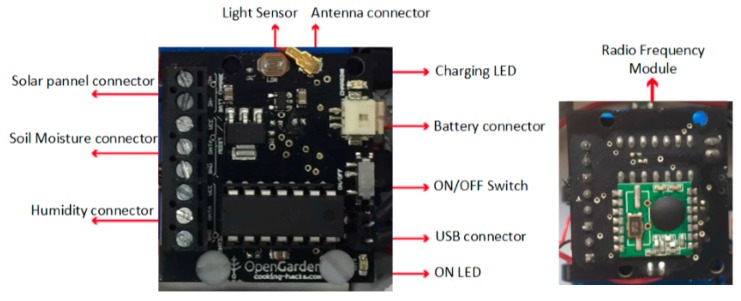
OpenGarden Slave Node functionality board [42].

The slave nodes are based on the use of OpenGarden Node Shield. As opposed to the central node, this board is simple, only in charge of connectivity with the different crop sensors to be monitored. Figure 4 shows a diagram of the connectivity board.

**Figure 5:**
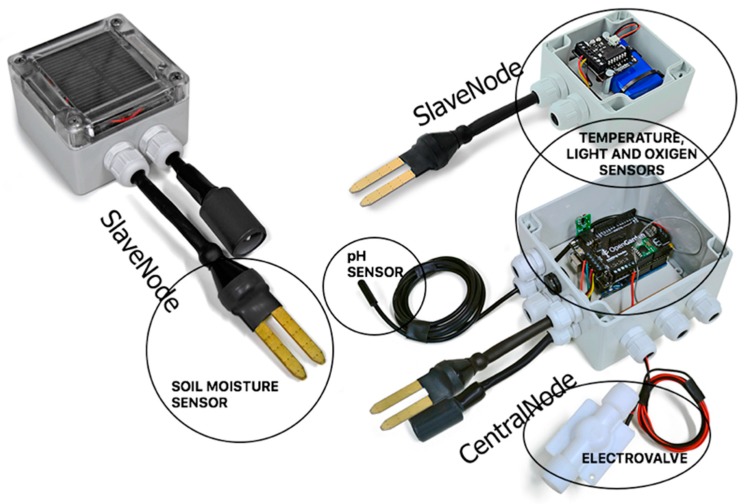
Look and feel of the devices.

As the functioning should be autonomous, this board also has a solar panel that is continually charging a lithium battery. The result of the complete assembly of the slave node and the central node are shown in Figure 5.

One of the novelties of the system is the use of the light agents that are embedded in the nodes [24]. The light agents are especially designed for implementation in devices and sensors with limited resource constraints. In this case the sensors have limited resources and are therefore embedded in software agents that can communicate with the PANGEA architecture; to reduce computational costs, a simple communication protocol is used. The central node contains an agent that retrieves information for the agents in the Slave Node, this communication is made using the 433 MHz radio frequency. The central node sends the information to the server with REST and the information is made available to the other agent in the virtual organization so that it can be displayed by different devices.

### 4.1. Irrigation System Based on Fuzzy Logic

**Figure 6:**
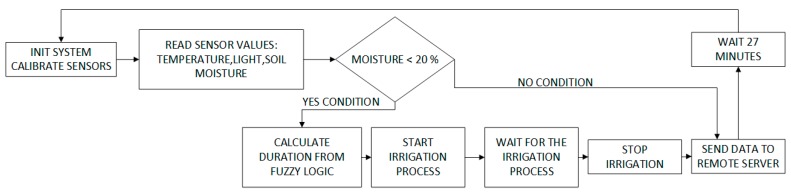
Process workflow: the main loop introduces a time delay in the whole process. Once the sensors are calibrated and measures obtained, the moisture level is checked. If the humidity crosses a given threshold, data is sent to the server. Otherwise, the input values of the sensors are sent to the fuzzy logic algorithm and the irrigation process is triggered.

As mentioned in Section 3, the virtual *Information Fusion* organization aims to adapt and process information from each of the sensors. This organization merges the information collected from each of the individual sensors, and estimates the flow of necessary irrigation at each moment. For the fusion of information from the sensors and the establishing of the volume of water for irrigation, fuzzy logic is used as explained in this section. The reason for using fuzzy logic as opposed to other alternatives, such as Bayes, is because we want to establish a continuous irrigation level and not by categories [43]. A diagram of the flowchart detailing the procedures that take place in the Information Fusion organization in provided in Figure 6. Readers may check [3] for further information.

The workflow of the irrigation process is described in the following paragraphs:

After the initial installation and activation carried out by the farmer, our system begins an auto-evaluation process where it verifies the condition of the installed sensors. If some type of interconnection error occurs, it is reported to the user through an alert. The nodes have an initial connection time of 10 s to connect to the master node. Once the sensors are connected to the WSN and the link with the central node is established via radio, they are ready to collect measurement data.

**Figure 7:**
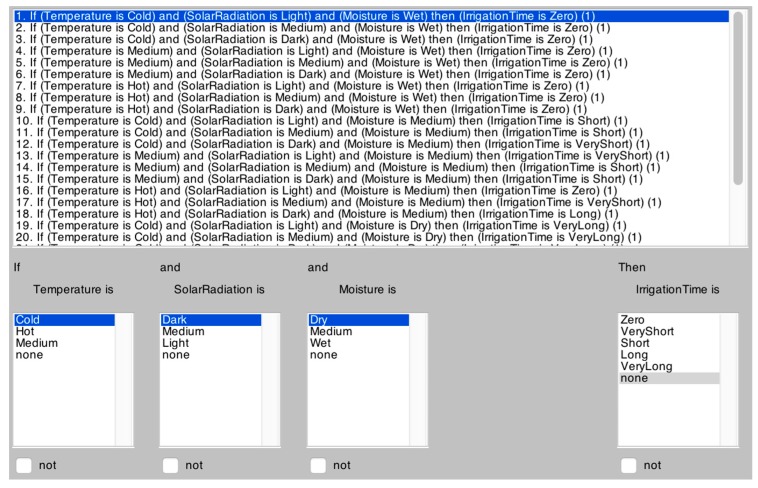
Rules provided by a human expert.

When the sensors are launched correctly, each one collects data according to the sampling frequency established by the user. Each of the slave nodes is in charge of collecting different measurements from the sensors, converting them into a format that can be read by a human and transmitting them to the central node. Each measure received by the central node is compared with the previous measures and the state of the humidity sensor is analyzed. If the value of the sensors is above 20%, all the information is sent to the central server with the aim of visualizing these data in the developed applications. However, if the humidity sensor displays a value that is below 20%, although all the necessary irrigation conditions are supplied (temperature, radiation, light, humidity) the required irrigation time will also have to be determined apart from sending the data. The sensors’ measures are used as input variables for the fuzzy logic system which measures the exact irrigation time. The empirical rules used by the fuzzy logic system, have been established by a farmer who is an experienced tomato cultivator. These rules can be seen in Figure 7.

Moreover, the server continually stores data in the database; the values collected by the sensors as well as the decisions taken by fuzzy logic, enabling the user to access all this information remotely through the application designed in Section 4.2.

As shown in Figure 6, when the crops are being irrigated, sensor readings cannot be taken until 27 min after the irrigation started, this is due to the effects of transpiration. If soil is watered under conditions of extreme heat, the water will evaporate and the ground will not dampen immediately, resulting in an incorrect reading. The 27 min period allows the sensor to retrieve the correct value for subsoil humidity. This time window is fixed and was calculated by performing evaporation tests during the month of July in the town of Salamanca, Spain. It is possible to find literature on how to calculate time dynamically [44,45], however it is not the focus of this work.

The goal of the fuzzy logic based algorithm [3,46] is to determine the volume of water and the duration of irrigation (opening of electric valve) required in each case. Knowledge rules are established for the humidity sensor in three situations: when the sensor is wet, when the sensor is partly wet and finally when the sensor is dry. Table 2 shows the irrigation time for each case.

To determine the reduction of uncertainty levels that comes with the inclusion of these three variables, an analysis of irrigation estimations is carried out through the Bayes application, on the basis of the use of these variables. The accuracy percentages obtained are listed in Table 3. As can be seen, when the three variables are used the accuracy increases. From this we can conclude that the three variables used are important in reducing the uncertainty when estimating the level of irrigation. We should also highlight that when using other classifiers, based on decision trees, such as J48 accuracy rises to 100%, however Bayes has been used given that it is the alternative to fuzzy logic listed in [43].

**Figure 8:**
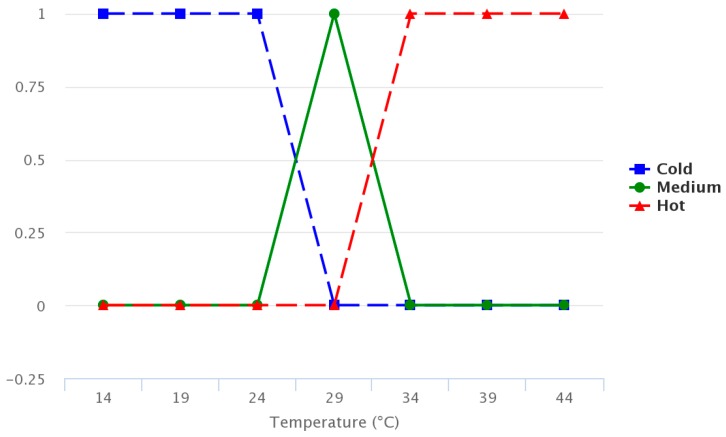
Temperature inference rule.

**Figure 9:**
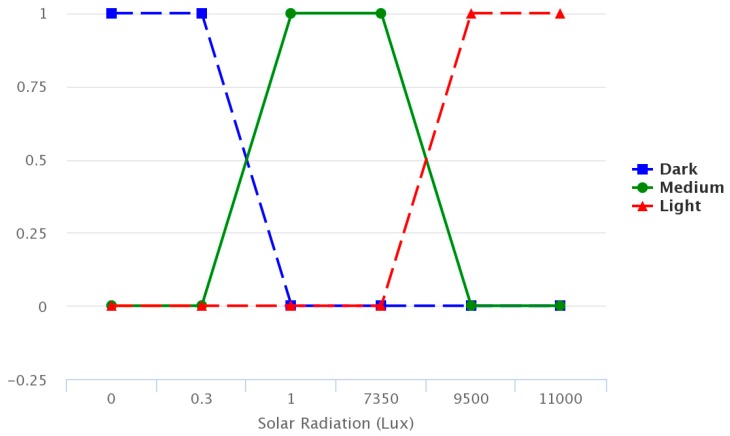
Solar radiation inference rule.

**Figure 10:**
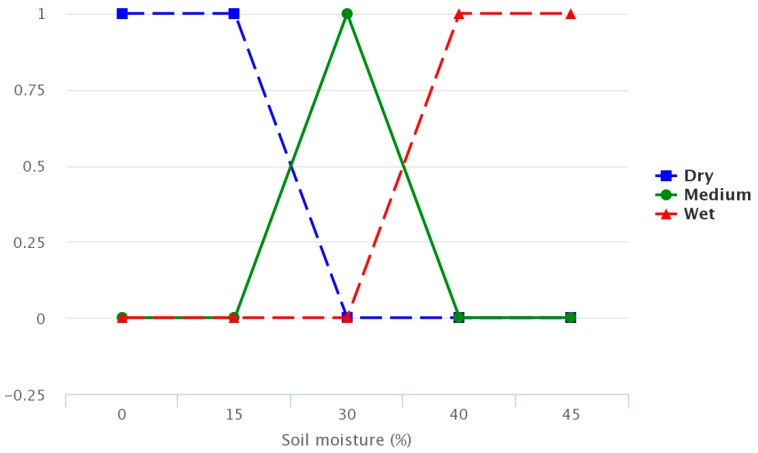
Soil inference rule.

The figures below provide information regarding the membership functions that have been used by the fuzzy sets. Figure 8, Figure 9 and Figure 10 represent the inference rules for temperature, solar radiation and soil moisture, respectively.

**Figure 11:**
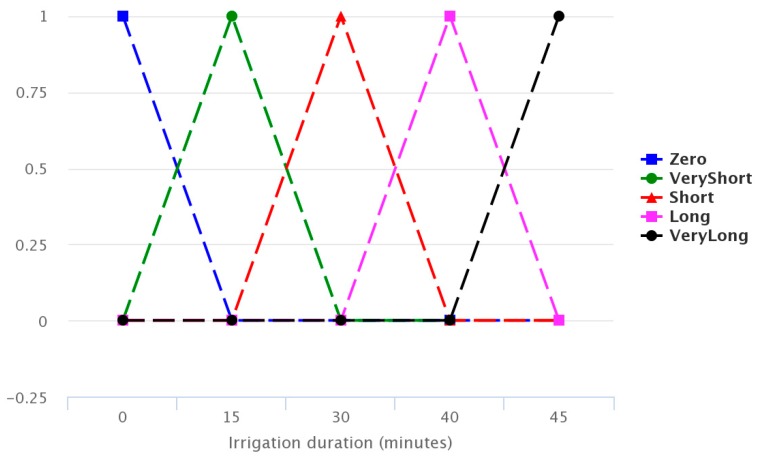
Irrigation time estimate.

Finally, Figure 11 combines the inference rules, showing the estimated irrigation time.

**Figure 12:**
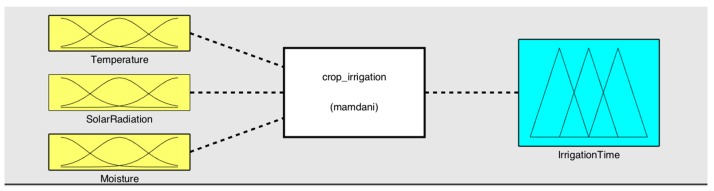
Inputs/output.

The algorithm chosen in this work is based on a Mamdani system [3], in which the membership functions are trapezoidal. The reason for using this fuzzy system is that the library [47], which allows one to develop applications with fuzzy logic in microcontrollers based on the ATmega328p chipset, is the only one that possesses the Mamdani fuzzy system. In addition, the Takagi-Sugeno method is less intuitive and more computationally complex. While the defuzzification process can be done using different methods, the centroid technique method [48,49] was selected in this case. The fuzzy logic system was designed with MATLAB software. Figure 12 shows the general scheme.

**Figure 13:**
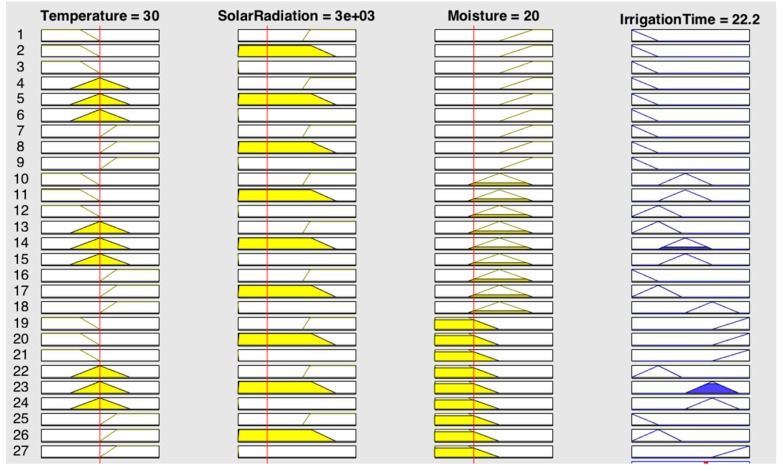
Output simulation.

Figure 13 shows the output produced by the fuzzy system, when the value of temperature is 30 °C, solar radiation is 3000 lux and the percentage of the subsoil moisture sensors is 20%.

**Figure 14:**
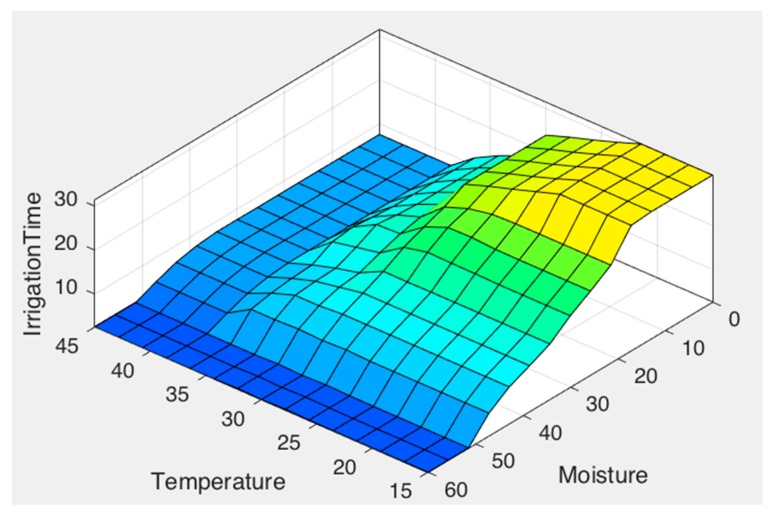
Relation between temperature and moisture.

**Figure 15:**
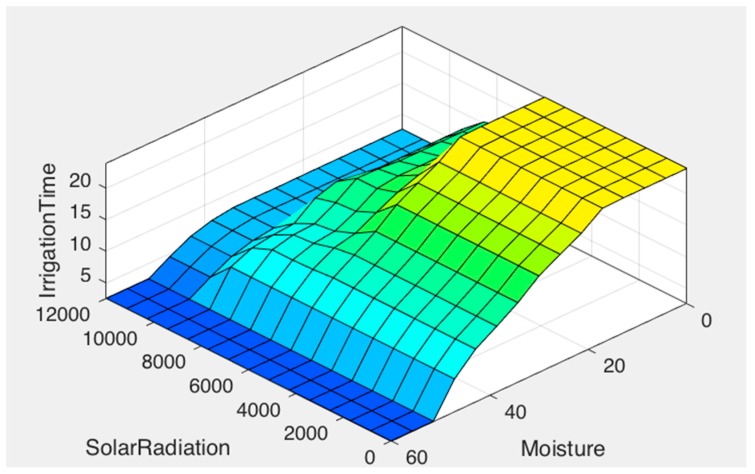
Relation between solar radiation and moisture.

As shown in Figure 14, when the temperature is high, irrigation time is completely determined by it, this helps to avoid water evaporation. In addition, Figure 15 shows how irrigation time increases as the humidity sensor approaches dry values and brightness.

Once the defuzzification process has been carried out, the following preliminary conclusions were obtained:

- The *subsoil moisture sensor* provides the most important system information; it measures the moisture of the subsoil and indicates when it is necessary to activate the irrigation mechanism. In addition, it estimates the amount of water needed.
- The *outdoor temperature sensor* measures the outside temperature. If the temperature is high, this sensor prevents the watering process that, if activated, would simply result in water evaporation and unnecessary water wastage.
- The *solar radiation sensor* is as necessary as the outside temperature sensor, since sunlight causes water to evaporate.

### 4.2. Platform Display

**Figure 16:**
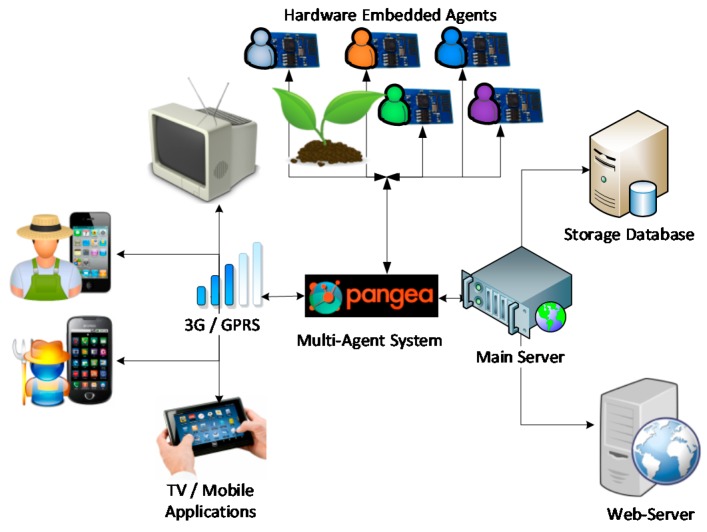
Scheme platform.

All irrigation systems must be controlled and monitored remotely. This section describes the physical connectivity of the system. The developed system can be deployed in any geographical location, provided that there is a data connection (Wi-Fi/3 G GSM) allowing the data from the sensors to be sent to a platform that resides on a central server. All wireless sensors have batteries that are continuously charged by solar energy. The objective of this section is to describe how individuals who are not familiar with technology could use the proposed system to check the state of their plants in real time. The overall architecture, including the screen display, is shown in Figure 16.

**Figure 17:**
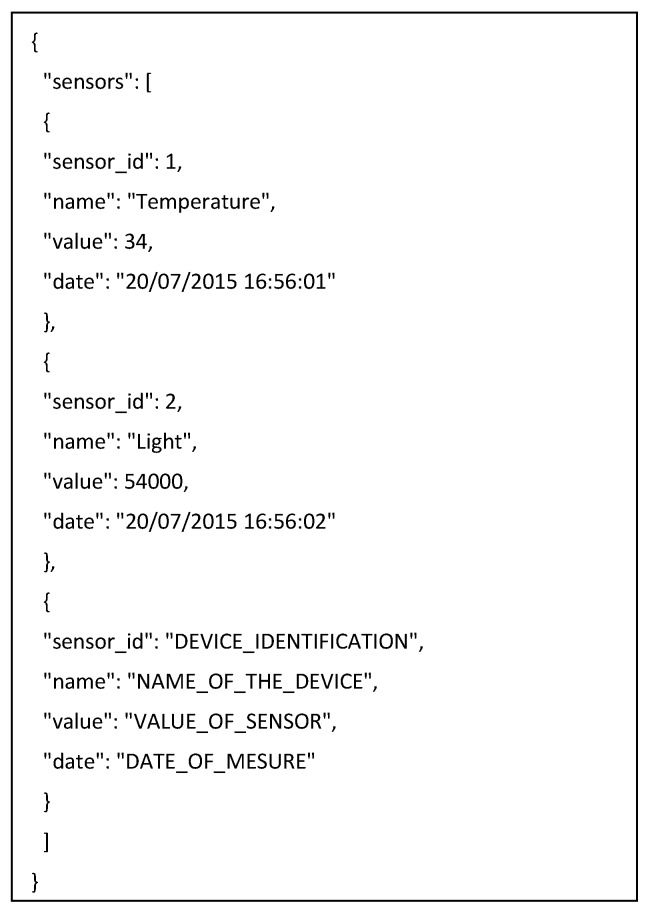
Message structure.

The agents embedded in the sensor network send the collected data periodically to organizations of agents located on the central server. This data can be kept and subsequently displayed. The communication between agents is done via RESTful web services, which allows for minimal battery consumption and high speed. Data exchange is done through JSON frames. This format was chosen because the data can be parsed by agents that are embedded in limited computing devices and very little time is required to plot the information. In Figure 17 below we give an example of the information structure.

**Figure 18:**
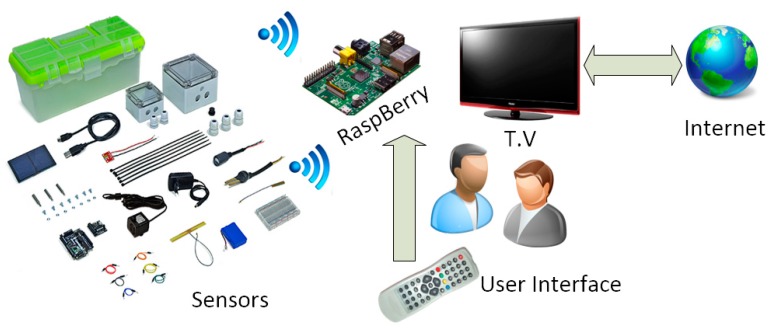
Remote control platform of the irrigation system.

The most innovative feature presented in this Section is the use of a display agent installed on a Raspberry PI device, which allows us to connect to any type of Smart TV browser that has an HDMI adapter. The goal is to provide all users, particularly elderly farmers, with a visualization agent which will allow them to view the condition of their crops easily and from their own home. To do this, the architectural design was implemented, as shown in the figure below (Figure 18).

**Figure 19:**
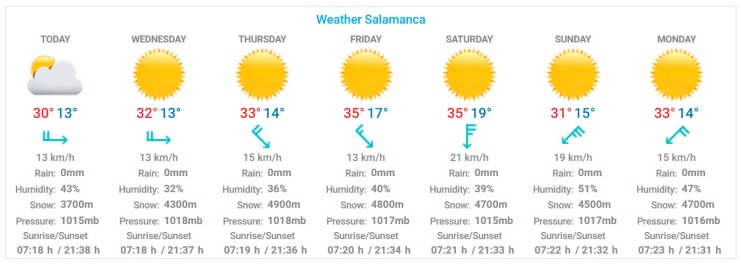
Smart TV application that shows the weather forecast.

**Figure 20:**
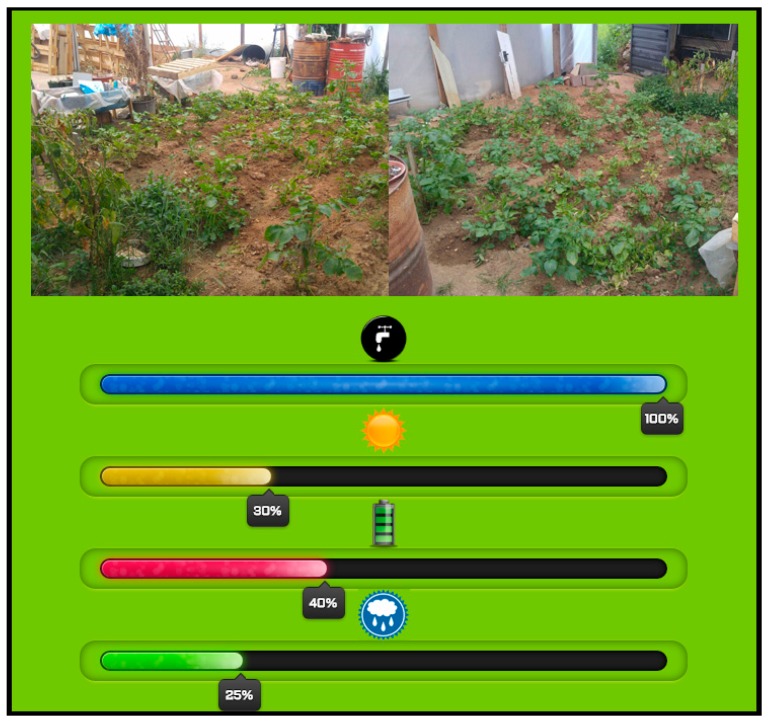
User interface screen capture with information from the different sensors displayed on the TV platform.

The display agent allows the user to interact with the system via the TV remote control. The first time that the farmer opens the application, he has to carry out an initial configuration, in which he chooses the type of crop and its geographic location. In this way, we preload the initial irrigation configuration which the user can modify according to their preferences. Using an infrared sensor, the user will be able to monitor and control the state of the different sensors in a web environment. In addition, the display agent alarms the user if one of the system sensors fails, even if the user is watching TV, a warning will display on the screen. Figure 19 and Figure 20 show a general view of the user interface of the proposed system, which uses a normal television to check the condition of plants.

In Figure 20, we can see the place where the system has been implemented, the farm has a size of 250 m^2^ and was loaned to us by a farmer for the purpose of this case study. The farm in this case study does not have large dimensions, this is because we wanted to avoid economic loss if the result happened to be negative and growth would be affected.

For now, the cameras are simply used to provide the user with a snapshot of the system at a given moment; in future works, however, we are planning to add a camera-based monitoring system to our architecture.

## 5. Results

This work presented the development of an intelligent system based on WSN that monitors and automates crop irrigation in an easy and economical way. The multi-agent architecture chosen to develop the case study is based on PANGEA, due to its ease of use and the ability to deploy agent drivers on computationally limited devices. The low cost of the proposed system (100 €/250 m^2^) is a key factor, it makes it an accessible tool to the majority of farmers who cannot afford to implement existing solutions.

The location chosen to test and validate the system was a rural garden located in the outskirts of Salamanca, in the town of Roblija de Cojos. The tomato crop in the garden had a WSN composed of various sensors which measured soil moisture, soil temperature, external temperature, light, rain and wind. The nodes were evenly distributed, with one node placed every 5 m^2^. Since the garden has an area of 250 m^2^, slave nodes and a central node, which coordinate communication, were also installed. The main characteristics of the field included: clay soil, no crop yield in the last five years, fallow land, no presence of nematodes.

Table 4 provides a comparison between the costs of the commercial systems that have been described in the Background Section 2 and the system proposed in this work. The price of these devices has been calculated on the basis of the configuration that they would require for the case study conducted in this work; a field of 250 m^2^. The calculated costs do not include additional teleoperator expenses for 3G/GPRS connection. The chosen systems do not have any installation costs since they are self-installing systems and the procedure can be carried out by the user. 250 m^2^ is established as the baseline size, which is the minimum field range within which the system is useful and its measurements are conclusive. The implementation costs of the other systems are also calculated for a similar area, between 150 and 250 m^2^. As can be seen, the proposed system has a smaller cost in comparison to the rest of commercial devices, even though the sensitizing areas are broader.

The type of tomato chosen for the testing process was the Pyros tomato. The Pyros tomato is a productive variety, of indeterminate growth, with a similar precocity to the Montfavet variety, resistant to cracking, with an average weight of 130 g, eye-catching green color, and resistant to *Verticillium*. Surface drip irrigation, 4 L/h of flow drip with a planting framework of 80 cm between rows and 25 cm between plants, with a plant density of 40 plants/100 m^2^, in a single line of cultivation.

**Figure 21:**
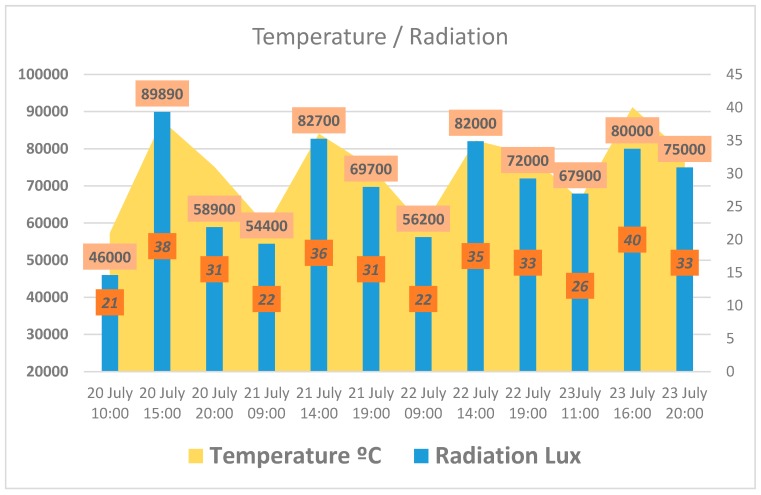
Relationship between temperature and solar radiation at different times of day.

**Figure 22:**
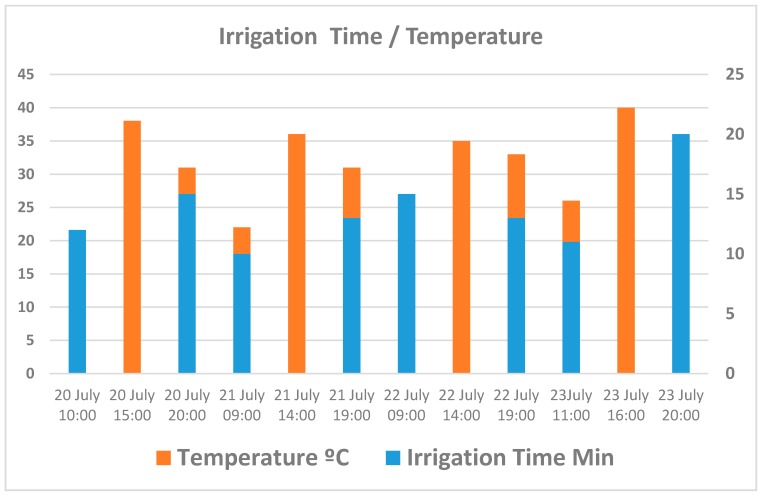
Relationship between the watering time and temperature.

Above we present the diverse results obtained in the case study. Figure 21 shows the different temperature and radiation measurements taken at different times of the day. Figure 22 presents the relationship between temperature and duration of irrigation. We see that at times of extreme temperatures, the irrigation system was not activated in order to avoid water evaporation.

**Figure 23:**
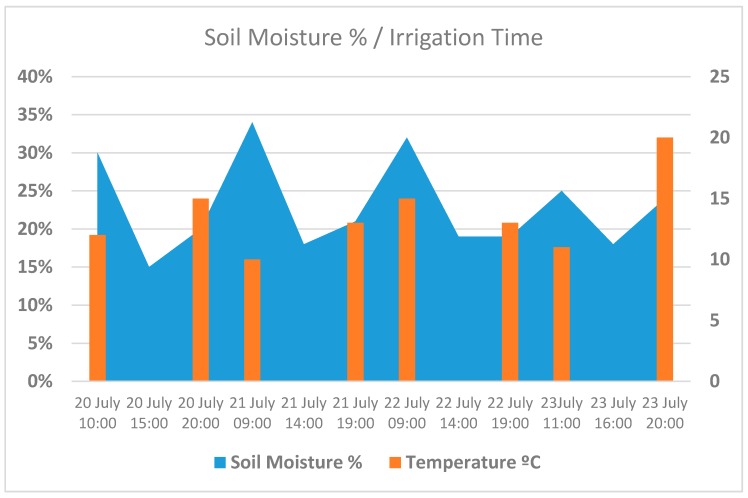
Relationship between moisture and watering time.

**Figure 24:**
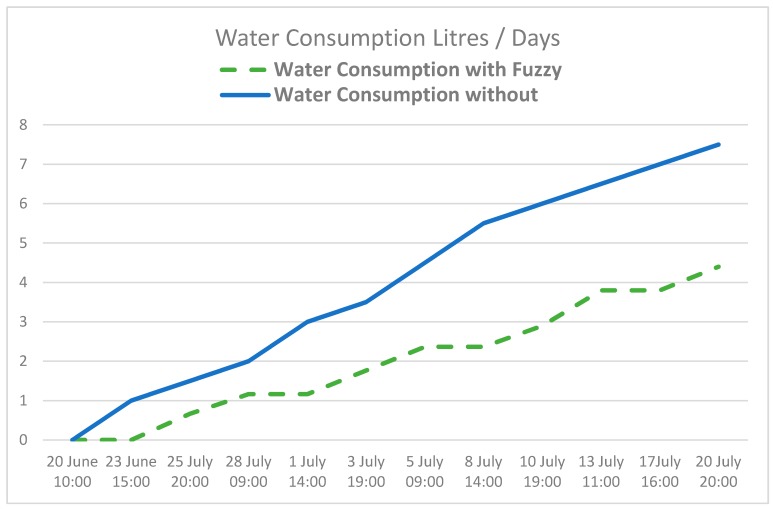
Total water consumption in the system using the fuzzy logic system.

Figure 23 shows how humidity drops to a minimum value at the hottest moments of the day. After irrigation is begun, we can immediately see that humidity increases. Figure 24 displays water consumption levels for an area of tomato crop measuring 50 m^2^, using conventional programmed irrigation compared with the system proposed in this article.

**Figure 25:**
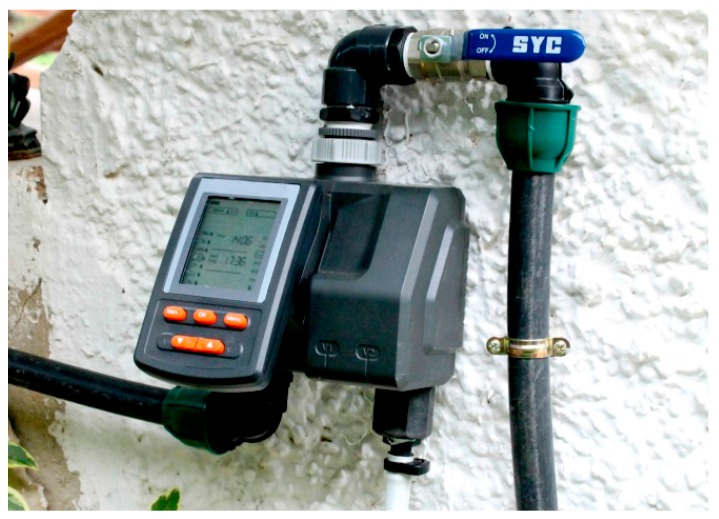
Commercial device used for comparison with the conducted case study.

The results of the case study have been compared with the traditional automatically programmed irrigation system. Concretely, an irrigation programming device has been used, it is called Orbit B-Hyve [32] with a cost of 130 $. A description and an image are included below (Figure 25).

The crop was always irrigated at dawn, using 1 L of water, and in the evening, using 0.5 L of water. As shown in the image, water consumption in a traditional system is linear, and does not consider any external factors, meaning that the amount of water used for irrigation is always the same and occurs at the same hours of the day. However, the use of the proposed architecture guarantees that the precise amount of water is used, depending on the sensors values and the weather. Both systems were evaluated during 30 days, in comparison to the traditional system, 37% were achieved with the traditional system.

As mentioned before, the conventional system was applied to an area of 10 m^2^ while the new system was used on an area of 50 m^2^. The location was contiguous and there was no difference between the crops. Although less water was used with the proposed system, crop production per square meter is very similar; the proposed system 4.73 kg/m^2^ tomatoes were collected as opposed to the 4.65 kg/m^2^ of the commercial one. Production is not very high, given that it follows a normal cycle; crops are planted in January and harvested in summer. In other regions, where crops are grown in greenhouses, up to 10 kg/m^2^ can be obtained.

## Figures and Tables

**Table 1:** Monitoring variables.

Variable	I/O	Description
LightAgent	I	Obtains the brightness of the environment.
TempAgent	I	Responsible for measuring the environmental temperature.
HumidityAgent	I	Agents whose primary function is to measure the moisture in the air.
ElectroValveAgent	I	Responsible for increasing or decreasing the water flow.
OxygenAgent	I	Obtains the level of oxygen in the air.
SoilMostureAgent	I	Measures the degree of moisture in the subsoil
WaterAgent	I	Indicates the amount of water in the tank.
OrganizationAgent	I/O	Responsible for the communication between the different agents.

**Table 2:** Rules for irrigation estimation time.

	Solar Radiation (Lux)			
Humidity	Light	Medium	Dark	Temperature
Wet	No Water	No Water	No Water	Cold
	No Water	No Water	No Water	Medium
	No Water	No Water	No Water	Hot
Half-Wet	Short	Short	Very Short	Cold
	Very Short	Short	Short	Medium
	No Water	Very Short	Long	Hot
Dry	Very Long	Very Long	Very Long	Cold
	Very Short	Long	Long	Medium
	No Water	Very Short	Very Long	Hot

**Table 3:** Accuracy percentage (calculated with Bayes) depending on the irrigation levels, according to the variables indicated in Table 2.

Sensors	Accuracy
Humidity	62.96%
Light	40.74%
Temperature	40.74%
Humidity/light	62.96%
Humidity/temperature	74.07%
Light/temperature	40.74%
Humidity/light/temperature	81.48%

**Table 4:** Comparative between the proposed solution and the main commercial solutions currently available on the market.

NAME	INCLUDED SENSORS	MAXIMUM ZONES	CONNECTIVITY	SCALABLE (PRICE)	SYSTEM PRICE
**AIFRO WATERECO [26]**	Humidity and temperature	16	Wi-Fi	No	180 $
**BLOSSOM [27]**	Humidity and temperature	12	Wi-Fi	No	200 $
**BLUESPRAY [28]**	Humidity, light, oxygen and water	16	Wi-Fi & Ethernet	Yes (80 $)	250 $
**GREENIQ [29]**	Humidity and light	6	Wi-Fi & Ethernet	No	212 $
**IRRIGATIONCADDY [30]**	Humidity	10	Wi-Fi & Ethernet	No	175 $
**LONO [31]**	Humidity, light and oxygen	20	Wi-Fi & Bluetooth	No	250 $
**ORBIT B-HYVE [32]**	Humidity	12	Wi-Fi	No	130 $
**RACHIO SMART SPRINKLER CONTROLLER [33]**	Humidity, temperature and water	8	Wi-Fi	Yes (60 $)	200 $
**RAINMACHINE [34]**	Humidity, soil moisture, temperature, water	16	Wi-Fi	Yes (80–110 $)	300 $
**SPRUCE IRRIGATION [35]**	Humidity, soil moisture and temperature	16	-	No	170 $
**RAINCOMMANDER [36]**	Humidity and light	12	Wi-Fi	No	250 $
**PROPOSED SYSTEM**	Humidity, soil moisture, temperature, light, water and oxygen	256	Wi-Fi, GPRS, Ethernet & RF	Yes (15 $)	100 $
